# Methyl 12-hydroxy-10-[1-(4-meth­oxy­phen­yl)-2-oxo-3-phen­oxy­azetidin-4-yl]-11-oxa-3-aza­hexa­cyclo­[11.7.1.0^2,9^.0^2,12^.0^3,7^.0^17,21^]henicosa-1(20),13,15,17(21),18-penta­ene-9-carboxyl­ate

**DOI:** 10.1107/S1600536812045795

**Published:** 2012-11-10

**Authors:** Sivasubramanian Suhitha, Thothadri Srinivasan, Raju Rajesh, Raghavachary Raghunathan, Devadasan Velmurugan

**Affiliations:** a Centre of Advanced Study in Crystallography and Biophysics, University of Madras, Guindy Campus, Chennai 600 025, India; b Department of Organic Chemistry, University of Madras, Guindy Campus, Chennai 600 025, India

## Abstract

In the title compound, C_37_H_34_N_2_O_7_, both pyrrolidine rings adopt envelope conformations. The β-lactam ring is close to planar (r.m.s. deviation = 0.0395 Å) and makes a dihedral angle of 83.35 (15)° with the furan ring. The O atom attached to the β-lactam ring deviates by 0.187 (2) Å from the mean plane of the ring. The β-lactam ring makes dihedral angles of 14.90 (15) and 27.72 (17)° with the meth­oxy­phenyl and phenyl rings, respectively. The crystal packing features C—H⋯O hydrogen bonds.

## Related literature

For general background and therapeutic applications of β-lactams, see: Banik & Becker (2000 ▶); Brakhage (1998 ▶). For a related structure, see: Sundaramoorthy *et al.* (2012 ▶).
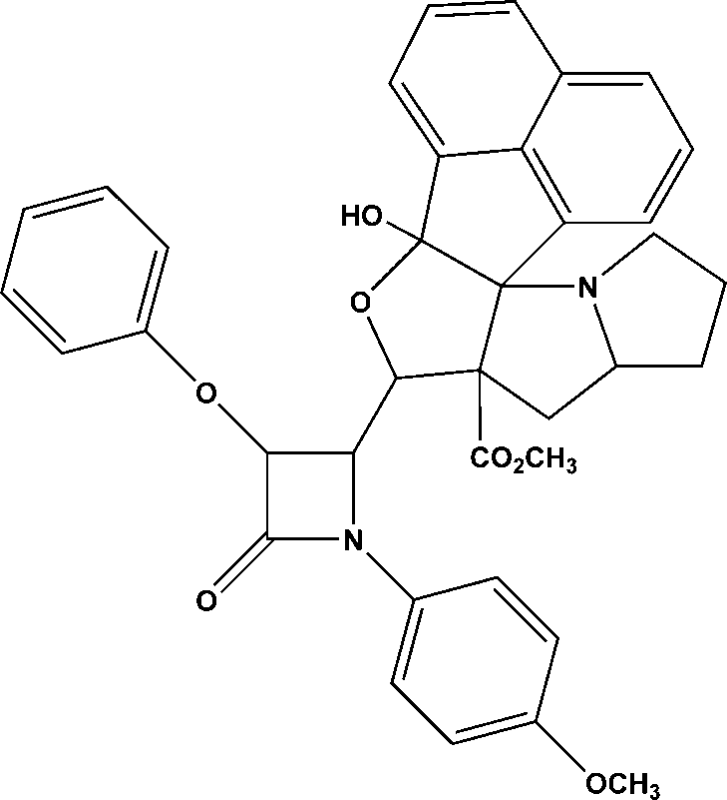


## Experimental

### 

#### Crystal data

C_37_H_34_N_2_O_7_
*M* *_r_* = 618.66
Orthorhombic, 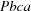

*a* = 9.6545 (11) Å
*b* = 20.363 (2) Å
*c* = 31.804 (3) Å
*V* = 6252.5 (11) Å^3^
*Z* = 8
Mo *K*α radiation
μ = 0.09 mm^−1^
*T* = 293 K
0.35 × 0.30 × 0.25 mm

#### Data collection

Bruker SMART APEXII area-detector diffractometer
Absorption correction: multi-scan (*SADABS*; Bruker, 2008 ▶) *T*_min_ = 0.969, *T*_max_ = 0.978
26528 measured reflections
5614 independent reflections
3210 reflections with *I* > 2σ(*I*)
*R*_int_ = 0.062

#### Refinement

*R*[*F* ^2^ > 2σ(*F* ^2^)] = 0.047
*wR*(*F* ^2^) = 0.127
*S* = 1.00
5614 reflections
421 parameters
H atoms treated by a mixture of independent and constrained refinement
Δρ_max_ = 0.14 e Å^−3^
Δρ_min_ = −0.21 e Å^−3^

### 

Data collection: *APEX2* (Bruker, 2008 ▶); cell refinement: *SAINT* (Bruker, 2008 ▶); data reduction: *SAINT*; program(s) used to solve structure: *SHELXS97* (Sheldrick, 2008 ▶); program(s) used to refine structure: *SHELXL97* (Sheldrick, 2008 ▶); molecular graphics: *ORTEP-3* (Farrugia, 2012) ▶; software used to prepare material for publication: *SHELXL97* and *PLATON* (Spek, 2009 ▶).

## Supplementary Material

Crystal structure: contains datablock(s) global, I. DOI: 10.1107/S1600536812045795/pv2604sup1.cif

Structure factors: contains datablock(s) I. DOI: 10.1107/S1600536812045795/pv2604Isup2.hkl

Supplementary material file. DOI: 10.1107/S1600536812045795/pv2604Isup3.cml

Additional supplementary materials:  crystallographic information; 3D view; checkCIF report

## Figures and Tables

**Table 1:** Hydrogen-bond geometry (Å, °)

*D*—H⋯*A*	*D*—H	H⋯*A*	*D*⋯*A*	*D*—H⋯*A*
C33—H33⋯O4^i^	0.93	2.49	3.409 (3)	173
C37—H37*C*⋯O7^ii^	0.96	2.53	3.217 (3)	128

Symmetry codes: (i) 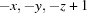; (ii) 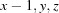.

**Fig. 1.**
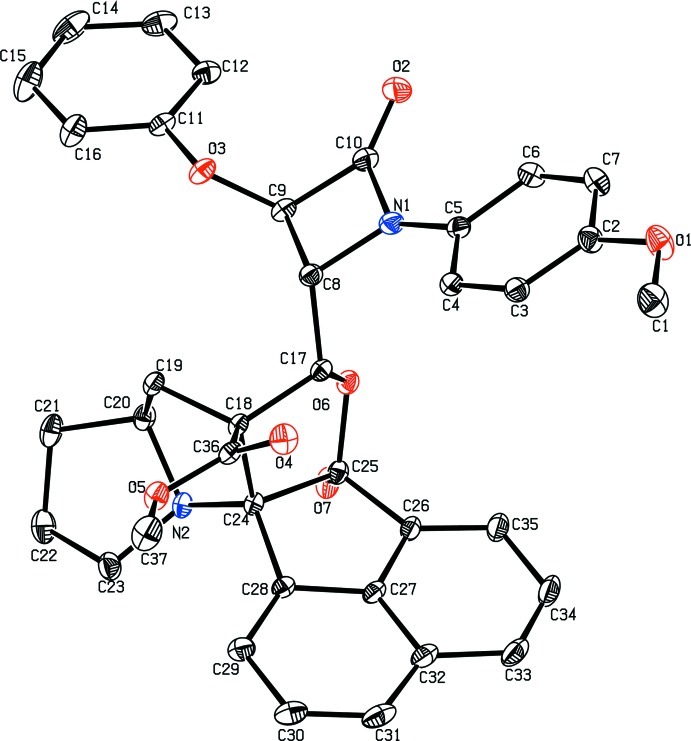
The molecular structure of the title compound with the atom numbering scheme. Displacement ellipsoids are drawn at the 30% probability level. H atoms were omitted for clarity.

**Fig. 2.**
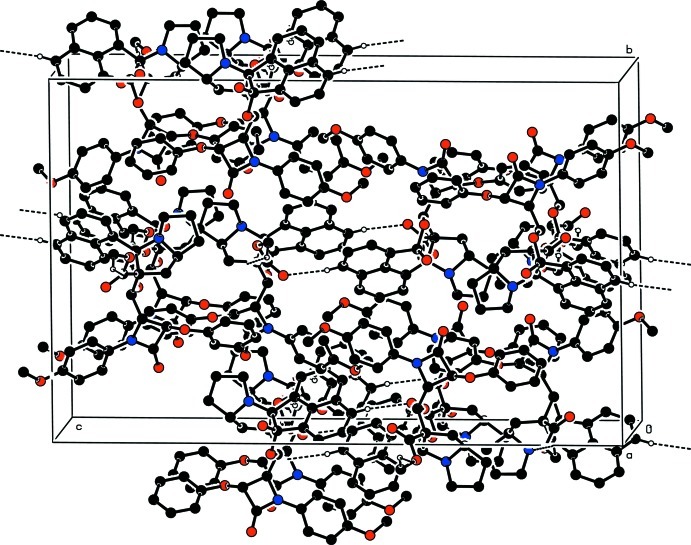
A view of the C—-H···O hydrogen bonds (dotted lines) in the crystal structure of the title compound viewed down b axis. H atoms non-participating in hydrogen-bonding were omitted for clarity.

## supplementary crystallographic information

### Comment

The role of β-lactam antibiotics is well known (Banik & Becker, 2000). The most commonly used β-lactam antibiotics for the therapy of infectious diseases are penicillin and cephalosporin (Brakhage, 1998). In view of potential applications of β-lactam derivatives, we have determined the crystal structure of the title compound and report it in this article.

In the title compound (Fig. 1), both pyrrolidine rings N2/C18–C20/C24 and N2/C20–C23 adopt C20- and C23-envelope conformations, respectively. The β lactam ring (N1/C8–C10) is essentially planar (rmsd = 0.0395 Å) and the O2 atom attached to it deviates by -0.187 (2)Å from its least-squares plane. The β lactam ring makes dihedral angles 14.90 (15)° and 27.72 (17)° with the methoxy phenyl and unsubstitued phenyl rings, respectively. The dihedral angle between the β lactam ring and the furan ring (O6/C17/C18/C24/C25) is 83.35 (15)°. The furan ring makes dihedral angles 81.84 (12)° and 72.24 (15)° with the two pyrrolidine rings. The dihedral angle between the furan ring and the cyclopentane ring (C24/C25/C26/C27/C28) is 71.56 (12)°. The bond distances and angles in the title compound agree very well with the corresponding bond distances and angles reported in a closely related compound (Sundaramoorthy *et al.*, 2012). The packing of the crystal structure is stabilised by intermolecular C—H···O hydrogen bonds (Tab. 1 & Fig. 2).

### Experimental

A solution of methyl 2-(hydroxy(1-(4-methoxyphenyl)-4-oxo-3-phenoxyazetidin -2-yl)methyl)acrylate (1.0 equiv.), acenaphthequinone (1.1 equiv.) and proline (1.1 equiv.) were refluxed in dry methanol. Completion of the reaction was evidenced by TLC analysis. The solvent was then removed under vacuum, diluted in dichloromethane and washed with brine and water. The organic layer was separated and removed and the residue subjected to column chromatography using ethyl acetate and hexane as an eluent (1:4) afforded the cycloadduct. The product was dissolved in chloroform and heated for two minutes. The resulting solution was subjected to crystallization by slow evaporation of the solvent for 48 hours resulting in the formation of single crystals.

### Refinement

The hydrogen atoms were placed in calculated positions with C—H = 0.93 Å to 0.98 Å and refined in the riding model with fixed isotropic displacement parameters:*U*_iso_(H) = 1.5*U*_eq_(methyl-C) and 1.2*U*_eq_(non-methyl C). The hydroxyl H-atom was located from a difference map and was allowed to refine freely.

### Crystal data

**Table:**

C_37_H_34_N_2_O_7_	*F*(000) = 2608
*M**_r_* = 618.66	*D*_x_ = 1.314 Mg m^−^^3^
Orthorhombic, *P**b**c**a*	Mo *K*α radiation, λ = 0.71073 Å
Hall symbol: -P 2ac 2ab	Cell parameters from 5614 reflections
*a* = 9.6545 (11) Å	θ = 1.3–25.3°
*b* = 20.363 (2) Å	µ = 0.09 mm^−^^1^
*c* = 31.804 (3) Å	*T* = 293 K
*V* = 6252.5 (11) Å^3^	Block, colourless
*Z* = 8	0.35 × 0.30 × 0.25 mm

### Data collection

**Table:**

Bruker SMART APEXII area-detector diffractometer	5614 independent reflections
Radiation source: fine-focus sealed tube	3210 reflections with *I* > 2σ(*I*)
Graphite monochromator	*R*_int_ = 0.062
ω and φ scans	θ_max_ = 25.3°, θ_min_ = 1.3°
Absorption correction: multi-scan (*SADABS*; Bruker, 2008)	*h* = −11→11
*T*_min_ = 0.969, *T*_max_ = 0.978	*k* = −22→24
26528 measured reflections	*l* = −32→38

### Refinement

**Table:**

Refinement on *F*^2^	Primary atom site location: structure-invariant direct methods
Least-squares matrix: full	Secondary atom site location: difference Fourier map
*R*[*F*^2^ > 2σ(*F*^2^)] = 0.047	Hydrogen site location: inferred from neighbouring sites
*wR*(*F*^2^) = 0.127	H atoms treated by a mixture of independent and constrained refinement
*S* = 1.00	*w* = 1/[σ^2^(*F*_o_^2^) + (0.0503*P*)^2^ + 1.3234*P*] where *P* = (*F*_o_^2^ + 2*F*_c_^2^)/3
5614 reflections	(Δ/σ)_max_ < 0.001
421 parameters	Δρ_max_ = 0.14 e Å^−^^3^
0 restraints	Δρ_min_ = −0.21 e Å^−^^3^

### Special details

**Table:**

Geometry. All esds (except the esd in the dihedral angle between two l.s. planes) are estimated using the full covariance matrix. The cell esds are taken into account individually in the estimation of esds in distances, angles and torsion angles; correlations between esds in cell parameters are only used when they are defined by crystal symmetry. An approximate (isotropic) treatment of cell esds is used for estimating esds involving l.s. planes.
Refinement. Refinement of F^2^ against ALL reflections. The weighted R-factor wR and goodness of fit S are based on F^2^, conventional R-factors R are based on F, with F set to zero for negative F^2^. The threshold expression of F^2^ > 2sigma(F^2^) is used only for calculating R-factors(gt) etc. and is not relevant to the choice of reflections for refinement. R-factors based on F^2^ are statistically about twice as large as those based on F, and R- factors based on ALL data will be even larger.

### Fractional atomic coordinates and isotropic or equivalent isotropic displacement parameters (Å^2^)

**Table:**

	*x*	*y*	*z*	*U*_iso_*/*U*_eq_	
C1	−0.1844 (4)	−0.29414 (16)	0.45652 (10)	0.0994 (11)	
H1A	−0.2716	−0.2931	0.4710	0.149*	
H1B	−0.1945	−0.3182	0.4307	0.149*	
H1C	−0.1553	−0.2501	0.4504	0.149*	
C2	−0.0580 (3)	−0.29816 (14)	0.52120 (9)	0.0668 (7)	
C3	−0.1206 (3)	−0.24169 (13)	0.53561 (8)	0.0614 (7)	
H3	−0.1869	−0.2206	0.5192	0.074*	
C4	−0.0846 (3)	−0.21631 (12)	0.57455 (7)	0.0580 (7)	
H4	−0.1261	−0.1778	0.5840	0.070*	
C5	0.0121 (3)	−0.24757 (12)	0.59942 (8)	0.0559 (6)	
C6	0.0725 (3)	−0.30568 (13)	0.58544 (9)	0.0688 (7)	
H6	0.1362	−0.3278	0.6022	0.083*	
C7	0.0370 (3)	−0.33014 (14)	0.54648 (9)	0.0742 (8)	
H7	0.0777	−0.3688	0.5370	0.089*	
C8	0.0036 (2)	−0.15872 (11)	0.65931 (7)	0.0531 (6)	
H8	−0.0971	−0.1585	0.6633	0.064*	
C9	0.0769 (2)	−0.18432 (12)	0.69942 (7)	0.0566 (6)	
H9	0.1632	−0.1609	0.7056	0.068*	
C10	0.1001 (3)	−0.24787 (14)	0.67485 (8)	0.0656 (7)	
C11	0.0410 (3)	−0.19644 (13)	0.77364 (7)	0.0569 (6)	
C12	0.1496 (3)	−0.23813 (13)	0.78101 (8)	0.0663 (7)	
H12	0.1945	−0.2591	0.7589	0.080*	
C13	0.1909 (3)	−0.24834 (16)	0.82211 (11)	0.0823 (9)	
H13	0.2639	−0.2768	0.8278	0.099*	
C14	0.1255 (4)	−0.2170 (2)	0.85429 (11)	0.1028 (13)	
H14	0.1525	−0.2250	0.8819	0.123*	
C15	0.0202 (4)	−0.1739 (2)	0.84622 (10)	0.1091 (13)	
H15	−0.0224	−0.1517	0.8683	0.131*	
C16	−0.0224 (3)	−0.16324 (17)	0.80586 (9)	0.0832 (9)	
H16	−0.0936	−0.1338	0.8003	0.100*	
C17	0.0505 (2)	−0.09544 (11)	0.63905 (7)	0.0465 (6)	
H17	0.0342	−0.0988	0.6087	0.056*	
C18	−0.0236 (2)	−0.03335 (11)	0.65529 (6)	0.0431 (5)	
C19	−0.0432 (2)	−0.02920 (12)	0.70346 (6)	0.0513 (6)	
H19A	−0.1280	−0.0060	0.7103	0.062*	
H19B	−0.0472	−0.0728	0.7157	0.062*	
C20	0.0831 (2)	0.00838 (13)	0.71975 (7)	0.0563 (6)	
H20	0.1602	−0.0219	0.7248	0.068*	
C21	0.0590 (3)	0.05173 (16)	0.75843 (8)	0.0771 (8)	
H21A	−0.0354	0.0472	0.7685	0.093*	
H21B	0.1220	0.0399	0.7809	0.093*	
C22	0.0859 (4)	0.12086 (16)	0.74376 (9)	0.0889 (9)	
H22A	0.1813	0.1334	0.7489	0.107*	
H22B	0.0251	0.1518	0.7579	0.107*	
C23	0.0555 (3)	0.11824 (13)	0.69729 (8)	0.0680 (7)	
H23A	−0.0433	0.1184	0.6918	0.082*	
H23B	0.0986	0.1545	0.6824	0.082*	
C24	0.0864 (2)	0.02290 (11)	0.64621 (6)	0.0433 (5)	
C25	0.2212 (2)	−0.01860 (12)	0.63326 (7)	0.0484 (6)	
C26	0.2334 (2)	−0.00885 (12)	0.58636 (7)	0.0523 (6)	
C27	0.1370 (2)	0.03869 (12)	0.57417 (7)	0.0516 (6)	
C28	0.0569 (2)	0.06374 (12)	0.60742 (7)	0.0486 (6)	
C29	−0.0358 (3)	0.11284 (13)	0.59889 (8)	0.0661 (7)	
H29	−0.0874	0.1318	0.6204	0.079*	
C30	−0.0519 (3)	0.13415 (15)	0.55704 (10)	0.0826 (9)	
H30	−0.1154	0.1673	0.5513	0.099*	
C31	0.0218 (4)	0.10812 (16)	0.52475 (9)	0.0859 (10)	
H31	0.0052	0.1223	0.4974	0.103*	
C32	0.1232 (3)	0.05987 (14)	0.53230 (8)	0.0669 (7)	
C33	0.2122 (4)	0.03017 (17)	0.50266 (9)	0.0858 (10)	
H33	0.2063	0.0419	0.4745	0.103*	
C34	0.3064 (4)	−0.01533 (17)	0.51499 (9)	0.0887 (10)	
H34	0.3646	−0.0337	0.4949	0.106*	
C35	0.3196 (3)	−0.03595 (14)	0.55723 (8)	0.0725 (8)	
H35	0.3853	−0.0671	0.5649	0.087*	
C36	−0.1586 (2)	−0.02405 (13)	0.63177 (7)	0.0500 (6)	
C37	−0.3568 (3)	0.04349 (18)	0.62279 (9)	0.0912 (10)	
H37A	−0.3327	0.0532	0.5942	0.137*	
H37B	−0.3997	0.0812	0.6353	0.137*	
H37C	−0.4200	0.0071	0.6235	0.137*	
N1	0.0482 (2)	−0.22067 (10)	0.63899 (6)	0.0595 (5)	
N2	0.11888 (18)	0.05505 (10)	0.68586 (5)	0.0501 (5)	
O1	−0.0823 (2)	−0.32569 (10)	0.48259 (6)	0.0937 (7)	
O2	0.1430 (2)	−0.30229 (10)	0.68253 (6)	0.0899 (6)	
O3	−0.01484 (17)	−0.18640 (9)	0.73418 (5)	0.0636 (5)	
O4	−0.19583 (18)	−0.05791 (10)	0.60292 (6)	0.0740 (5)	
O5	−0.23191 (15)	0.02664 (9)	0.64624 (5)	0.0635 (5)	
O6	0.19453 (14)	−0.08459 (8)	0.64559 (4)	0.0525 (4)	
O7	0.33797 (16)	0.00319 (10)	0.65408 (6)	0.0655 (5)	
H7A	0.303 (3)	0.0308 (14)	0.6745 (9)	0.090 (10)*	

### Atomic displacement parameters (Å^2^)

**Table:**

	*U*^11^	*U*^22^	*U*^33^	*U*^12^	*U*^13^	*U*^23^
C1	0.118 (3)	0.105 (3)	0.075 (2)	0.008 (2)	−0.029 (2)	−0.0208 (19)
C2	0.0727 (18)	0.0621 (18)	0.0657 (18)	−0.0071 (15)	−0.0012 (15)	−0.0129 (15)
C3	0.0669 (17)	0.0586 (17)	0.0587 (17)	−0.0053 (13)	−0.0017 (13)	−0.0016 (14)
C4	0.0671 (17)	0.0539 (16)	0.0529 (16)	−0.0033 (13)	0.0057 (13)	−0.0004 (13)
C5	0.0663 (16)	0.0493 (15)	0.0522 (16)	−0.0063 (13)	0.0047 (13)	0.0030 (12)
C6	0.0727 (18)	0.0570 (17)	0.077 (2)	0.0029 (14)	−0.0052 (15)	0.0009 (15)
C7	0.0778 (19)	0.0606 (18)	0.084 (2)	0.0053 (15)	−0.0018 (16)	−0.0166 (16)
C8	0.0601 (15)	0.0522 (15)	0.0469 (14)	−0.0038 (12)	−0.0009 (12)	0.0028 (12)
C9	0.0569 (15)	0.0662 (17)	0.0465 (15)	−0.0052 (12)	0.0012 (12)	0.0110 (13)
C10	0.0746 (18)	0.0639 (19)	0.0583 (18)	0.0046 (14)	0.0042 (14)	0.0121 (15)
C11	0.0588 (16)	0.0667 (17)	0.0451 (16)	−0.0158 (13)	−0.0017 (12)	0.0117 (13)
C12	0.0731 (18)	0.0641 (18)	0.0616 (18)	−0.0048 (14)	−0.0088 (14)	0.0124 (14)
C13	0.081 (2)	0.087 (2)	0.079 (2)	−0.0211 (17)	−0.0233 (18)	0.0271 (19)
C14	0.097 (3)	0.157 (4)	0.054 (2)	−0.048 (3)	−0.019 (2)	0.027 (2)
C15	0.094 (3)	0.179 (4)	0.055 (2)	−0.011 (3)	0.0026 (18)	−0.012 (2)
C16	0.0722 (19)	0.120 (3)	0.0577 (19)	0.0013 (18)	0.0054 (15)	0.0031 (18)
C17	0.0484 (13)	0.0535 (14)	0.0375 (13)	−0.0039 (11)	−0.0004 (10)	0.0025 (11)
C18	0.0391 (12)	0.0570 (14)	0.0330 (12)	−0.0001 (10)	0.0016 (9)	0.0036 (10)
C19	0.0510 (13)	0.0648 (16)	0.0382 (13)	0.0031 (11)	0.0058 (10)	0.0045 (11)
C20	0.0524 (14)	0.0747 (17)	0.0418 (14)	0.0066 (12)	−0.0042 (11)	−0.0020 (13)
C21	0.0775 (19)	0.113 (3)	0.0409 (16)	0.0027 (17)	−0.0011 (13)	−0.0145 (16)
C22	0.103 (2)	0.097 (2)	0.067 (2)	0.0008 (19)	0.0008 (17)	−0.0316 (18)
C23	0.0772 (18)	0.0650 (18)	0.0617 (17)	0.0004 (14)	0.0044 (14)	−0.0175 (14)
C24	0.0404 (12)	0.0527 (14)	0.0368 (13)	−0.0028 (10)	0.0015 (9)	−0.0021 (10)
C25	0.0416 (13)	0.0571 (16)	0.0465 (14)	−0.0047 (11)	0.0058 (10)	−0.0030 (12)
C26	0.0561 (14)	0.0570 (16)	0.0438 (14)	−0.0146 (12)	0.0117 (11)	−0.0082 (12)
C27	0.0610 (15)	0.0563 (16)	0.0373 (14)	−0.0198 (12)	0.0040 (11)	0.0013 (11)
C28	0.0492 (13)	0.0526 (15)	0.0439 (14)	−0.0098 (11)	−0.0004 (11)	0.0045 (11)
C29	0.0676 (17)	0.0652 (18)	0.0654 (18)	0.0000 (14)	0.0000 (14)	0.0128 (14)
C30	0.091 (2)	0.081 (2)	0.076 (2)	−0.0044 (17)	−0.0112 (18)	0.0299 (18)
C31	0.109 (3)	0.091 (2)	0.057 (2)	−0.029 (2)	−0.0130 (18)	0.0294 (17)
C32	0.085 (2)	0.0700 (19)	0.0459 (17)	−0.0277 (16)	0.0024 (15)	0.0067 (14)
C33	0.125 (3)	0.092 (3)	0.0404 (17)	−0.040 (2)	0.0145 (18)	−0.0012 (17)
C34	0.117 (3)	0.094 (3)	0.055 (2)	−0.027 (2)	0.0365 (18)	−0.0206 (18)
C35	0.0842 (19)	0.0754 (19)	0.0578 (18)	−0.0144 (15)	0.0238 (15)	−0.0135 (15)
C36	0.0443 (13)	0.0619 (17)	0.0439 (15)	−0.0061 (12)	0.0046 (11)	0.0079 (13)
C37	0.0504 (16)	0.142 (3)	0.081 (2)	0.0219 (17)	−0.0102 (14)	0.012 (2)
N1	0.0763 (14)	0.0545 (13)	0.0477 (13)	0.0003 (11)	−0.0036 (11)	0.0061 (11)
N2	0.0485 (11)	0.0630 (13)	0.0387 (11)	−0.0008 (9)	0.0011 (9)	−0.0083 (10)
O1	0.1088 (17)	0.0879 (15)	0.0843 (15)	0.0127 (12)	−0.0236 (12)	−0.0371 (12)
O2	0.1236 (17)	0.0733 (14)	0.0729 (14)	0.0277 (13)	−0.0007 (12)	0.0158 (11)
O3	0.0584 (10)	0.0863 (13)	0.0459 (10)	−0.0035 (9)	−0.0015 (8)	0.0165 (9)
O4	0.0688 (12)	0.0918 (14)	0.0613 (12)	−0.0018 (10)	−0.0224 (9)	−0.0095 (10)
O5	0.0418 (9)	0.0909 (13)	0.0579 (10)	0.0123 (9)	−0.0013 (8)	0.0041 (9)
O6	0.0442 (9)	0.0612 (11)	0.0520 (10)	0.0042 (8)	0.0053 (7)	0.0041 (8)
O7	0.0386 (9)	0.0956 (14)	0.0622 (12)	−0.0063 (9)	0.0009 (8)	−0.0150 (11)

### Geometric parameters (Å, º)

**Table:**

C1—O1	1.440 (3)	C19—H19A	0.9700
C1—H1A	0.9600	C19—H19B	0.9700
C1—H1B	0.9600	C20—N2	1.478 (3)
C1—H1C	0.9600	C20—C21	1.532 (3)
C2—O1	1.370 (3)	C20—H20	0.9800
C2—C3	1.378 (4)	C21—C22	1.506 (4)
C2—C7	1.383 (4)	C21—H21A	0.9700
C3—C4	1.386 (3)	C21—H21B	0.9700
C3—H3	0.9300	C22—C23	1.508 (4)
C4—C5	1.380 (3)	C22—H22A	0.9700
C4—H4	0.9300	C22—H22B	0.9700
C5—C6	1.392 (3)	C23—N2	1.470 (3)
C5—N1	1.416 (3)	C23—H23A	0.9700
C6—C7	1.379 (4)	C23—H23B	0.9700
C6—H6	0.9300	C24—N2	1.455 (3)
C7—H7	0.9300	C24—C28	1.515 (3)
C8—N1	1.482 (3)	C24—C25	1.605 (3)
C8—C17	1.510 (3)	C25—O7	1.381 (3)
C8—C9	1.549 (3)	C25—O6	1.423 (3)
C8—H8	0.9800	C25—C26	1.509 (3)
C9—O3	1.417 (3)	C26—C35	1.362 (3)
C9—C10	1.528 (4)	C26—C27	1.397 (3)
C9—H9	0.9800	C27—C28	1.406 (3)
C10—O2	1.208 (3)	C27—C32	1.406 (3)
C10—N1	1.363 (3)	C28—C29	1.369 (3)
C11—C12	1.369 (4)	C29—C30	1.408 (4)
C11—C16	1.372 (4)	C29—H29	0.9300
C11—O3	1.381 (3)	C30—C31	1.357 (4)
C12—C13	1.383 (4)	C30—H30	0.9300
C12—H12	0.9300	C31—C32	1.408 (4)
C13—C14	1.362 (5)	C31—H31	0.9300
C13—H13	0.9300	C32—C33	1.412 (4)
C14—C15	1.367 (5)	C33—C34	1.356 (4)
C14—H14	0.9300	C33—H33	0.9300
C15—C16	1.365 (4)	C34—C35	1.413 (4)
C15—H15	0.9300	C34—H34	0.9300
C16—H16	0.9300	C35—H35	0.9300
C17—O6	1.423 (2)	C36—O4	1.203 (3)
C17—C18	1.542 (3)	C36—O5	1.334 (3)
C17—H17	0.9800	C37—O5	1.458 (3)
C18—C36	1.514 (3)	C37—H37A	0.9600
C18—C19	1.546 (3)	C37—H37B	0.9600
C18—C24	1.589 (3)	C37—H37C	0.9600
C19—C20	1.530 (3)	O7—H7A	0.92 (3)
			
O1—C1—H1A	109.5	C22—C21—C20	105.3 (2)
O1—C1—H1B	109.5	C22—C21—H21A	110.7
H1A—C1—H1B	109.5	C20—C21—H21A	110.7
O1—C1—H1C	109.5	C22—C21—H21B	110.7
H1A—C1—H1C	109.5	C20—C21—H21B	110.7
H1B—C1—H1C	109.5	H21A—C21—H21B	108.8
O1—C2—C3	124.4 (3)	C21—C22—C23	103.7 (2)
O1—C2—C7	116.2 (3)	C21—C22—H22A	111.0
C3—C2—C7	119.4 (3)	C23—C22—H22A	111.0
C2—C3—C4	119.9 (3)	C21—C22—H22B	111.0
C2—C3—H3	120.0	C23—C22—H22B	111.0
C4—C3—H3	120.0	H22A—C22—H22B	109.0
C5—C4—C3	120.7 (2)	N2—C23—C22	101.1 (2)
C5—C4—H4	119.7	N2—C23—H23A	111.6
C3—C4—H4	119.7	C22—C23—H23A	111.6
C4—C5—C6	119.5 (2)	N2—C23—H23B	111.6
C4—C5—N1	119.8 (2)	C22—C23—H23B	111.6
C6—C5—N1	120.6 (2)	H23A—C23—H23B	109.4
C7—C6—C5	119.3 (3)	N2—C24—C28	119.95 (19)
C7—C6—H6	120.3	N2—C24—C18	108.12 (16)
C5—C6—H6	120.3	C28—C24—C18	114.73 (16)
C6—C7—C2	121.2 (3)	N2—C24—C25	106.54 (16)
C6—C7—H7	119.4	C28—C24—C25	103.42 (16)
C2—C7—H7	119.4	C18—C24—C25	102.06 (16)
N1—C8—C17	116.94 (19)	O7—C25—O6	108.61 (18)
N1—C8—C9	86.55 (17)	O7—C25—C26	111.61 (18)
C17—C8—C9	120.1 (2)	O6—C25—C26	114.24 (18)
N1—C8—H8	110.4	O7—C25—C24	111.69 (18)
C17—C8—H8	110.4	O6—C25—C24	106.23 (16)
C9—C8—H8	110.4	C26—C25—C24	104.32 (18)
O3—C9—C10	117.7 (2)	C35—C26—C27	119.9 (2)
O3—C9—C8	111.53 (19)	C35—C26—C25	131.8 (2)
C10—C9—C8	86.05 (18)	C27—C26—C25	108.28 (19)
O3—C9—H9	112.9	C26—C27—C28	114.1 (2)
C10—C9—H9	112.9	C26—C27—C32	122.6 (2)
C8—C9—H9	112.9	C28—C27—C32	123.3 (3)
O2—C10—N1	131.9 (3)	C29—C28—C27	118.4 (2)
O2—C10—C9	136.4 (3)	C29—C28—C24	133.2 (2)
N1—C10—C9	91.7 (2)	C27—C28—C24	108.1 (2)
C12—C11—C16	121.3 (2)	C28—C29—C30	119.0 (3)
C12—C11—O3	123.1 (2)	C28—C29—H29	120.5
C16—C11—O3	115.6 (2)	C30—C29—H29	120.5
C11—C12—C13	118.4 (3)	C31—C30—C29	122.5 (3)
C11—C12—H12	120.8	C31—C30—H30	118.8
C13—C12—H12	120.8	C29—C30—H30	118.8
C14—C13—C12	120.4 (3)	C30—C31—C32	120.5 (3)
C14—C13—H13	119.8	C30—C31—H31	119.7
C12—C13—H13	119.8	C32—C31—H31	119.7
C13—C14—C15	120.3 (3)	C27—C32—C31	116.2 (3)
C13—C14—H14	119.8	C27—C32—C33	116.3 (3)
C15—C14—H14	119.8	C31—C32—C33	127.5 (3)
C16—C15—C14	120.2 (3)	C34—C33—C32	120.5 (3)
C16—C15—H15	119.9	C34—C33—H33	119.7
C14—C15—H15	119.9	C32—C33—H33	119.7
C15—C16—C11	119.3 (3)	C33—C34—C35	122.6 (3)
C15—C16—H16	120.3	C33—C34—H34	118.7
C11—C16—H16	120.3	C35—C34—H34	118.7
O6—C17—C8	111.30 (18)	C26—C35—C34	118.1 (3)
O6—C17—C18	106.09 (17)	C26—C35—H35	121.0
C8—C17—C18	114.68 (18)	C34—C35—H35	121.0
O6—C17—H17	108.2	O4—C36—O5	123.2 (2)
C8—C17—H17	108.2	O4—C36—C18	124.2 (2)
C18—C17—H17	108.2	O5—C36—C18	112.5 (2)
C36—C18—C17	109.66 (18)	O5—C37—H37A	109.5
C36—C18—C19	112.16 (17)	O5—C37—H37B	109.5
C17—C18—C19	115.70 (18)	H37A—C37—H37B	109.5
C36—C18—C24	113.28 (17)	O5—C37—H37C	109.5
C17—C18—C24	102.72 (16)	H37A—C37—H37C	109.5
C19—C18—C24	102.86 (16)	H37B—C37—H37C	109.5
C20—C19—C18	105.37 (17)	C10—N1—C5	132.6 (2)
C20—C19—H19A	110.7	C10—N1—C8	95.04 (19)
C18—C19—H19A	110.7	C5—N1—C8	130.2 (2)
C20—C19—H19B	110.7	C24—N2—C23	121.23 (18)
C18—C19—H19B	110.7	C24—N2—C20	107.00 (17)
H19A—C19—H19B	108.8	C23—N2—C20	106.56 (18)
N2—C20—C19	105.13 (17)	C2—O1—C1	116.8 (2)
N2—C20—C21	104.5 (2)	C11—O3—C9	118.00 (18)
C19—C20—C21	116.0 (2)	C36—O5—C37	116.4 (2)
N2—C20—H20	110.3	C17—O6—C25	106.45 (16)
C19—C20—H20	110.3	C25—O7—H7A	103.4 (17)
C21—C20—H20	110.3		
			
O1—C2—C3—C4	−177.3 (2)	C35—C26—C27—C32	0.8 (4)
C7—C2—C3—C4	2.1 (4)	C25—C26—C27—C32	179.1 (2)
C2—C3—C4—C5	−0.9 (4)	C26—C27—C28—C29	176.9 (2)
C3—C4—C5—C6	−0.9 (4)	C32—C27—C28—C29	−2.3 (3)
C3—C4—C5—N1	179.6 (2)	C26—C27—C28—C24	−8.5 (3)
C4—C5—C6—C7	1.6 (4)	C32—C27—C28—C24	172.2 (2)
N1—C5—C6—C7	−179.0 (2)	N2—C24—C28—C29	−55.5 (3)
C5—C6—C7—C2	−0.3 (4)	C18—C24—C28—C29	75.9 (3)
O1—C2—C7—C6	178.0 (2)	C25—C24—C28—C29	−173.8 (3)
C3—C2—C7—C6	−1.5 (4)	N2—C24—C28—C27	131.1 (2)
N1—C8—C9—O3	−124.0 (2)	C18—C24—C28—C27	−97.5 (2)
C17—C8—C9—O3	116.7 (2)	C25—C24—C28—C27	12.8 (2)
N1—C8—C9—C10	−5.66 (17)	C27—C28—C29—C30	2.9 (4)
C17—C8—C9—C10	−125.0 (2)	C24—C28—C29—C30	−170.0 (2)
O3—C9—C10—O2	−59.7 (4)	C28—C29—C30—C31	−0.4 (4)
C8—C9—C10—O2	−172.0 (4)	C29—C30—C31—C32	−2.8 (5)
O3—C9—C10—N1	118.4 (2)	C26—C27—C32—C31	−179.9 (2)
C8—C9—C10—N1	6.14 (19)	C28—C27—C32—C31	−0.7 (4)
C16—C11—C12—C13	2.7 (4)	C26—C27—C32—C33	0.3 (4)
O3—C11—C12—C13	−175.3 (2)	C28—C27—C32—C33	179.5 (2)
C11—C12—C13—C14	−0.6 (4)	C30—C31—C32—C27	3.2 (4)
C12—C13—C14—C15	−1.6 (5)	C30—C31—C32—C33	−177.0 (3)
C13—C14—C15—C16	1.7 (6)	C27—C32—C33—C34	−1.1 (4)
C14—C15—C16—C11	0.3 (5)	C31—C32—C33—C34	179.2 (3)
C12—C11—C16—C15	−2.5 (4)	C32—C33—C34—C35	0.8 (5)
O3—C11—C16—C15	175.6 (3)	C27—C26—C35—C34	−1.0 (4)
N1—C8—C17—O6	−72.3 (2)	C25—C26—C35—C34	−178.8 (2)
C9—C8—C17—O6	30.2 (3)	C33—C34—C35—C26	0.3 (4)
N1—C8—C17—C18	167.29 (18)	C17—C18—C36—O4	−4.7 (3)
C9—C8—C17—C18	−90.2 (3)	C19—C18—C36—O4	−134.7 (2)
O6—C17—C18—C36	152.17 (16)	C24—C18—C36—O4	109.4 (3)
C8—C17—C18—C36	−84.6 (2)	C17—C18—C36—O5	176.02 (17)
O6—C17—C18—C19	−79.8 (2)	C19—C18—C36—O5	46.0 (3)
C8—C17—C18—C19	43.5 (3)	C24—C18—C36—O5	−69.9 (2)
O6—C17—C18—C24	31.4 (2)	O2—C10—N1—C5	7.8 (5)
C8—C17—C18—C24	154.71 (18)	C9—C10—N1—C5	−170.5 (2)
C36—C18—C19—C20	−140.6 (2)	O2—C10—N1—C8	171.8 (3)
C17—C18—C19—C20	92.5 (2)	C9—C10—N1—C8	−6.4 (2)
C24—C18—C19—C20	−18.6 (2)	C4—C5—N1—C10	155.1 (3)
C18—C19—C20—N2	32.5 (2)	C6—C5—N1—C10	−24.4 (4)
C18—C19—C20—C21	147.4 (2)	C4—C5—N1—C8	−3.9 (4)
N2—C20—C21—C22	−1.1 (3)	C6—C5—N1—C8	176.7 (2)
C19—C20—C21—C22	−116.3 (3)	C17—C8—N1—C10	128.5 (2)
C20—C21—C22—C23	26.2 (3)	C9—C8—N1—C10	6.35 (19)
C21—C22—C23—N2	−41.4 (3)	C17—C8—N1—C5	−66.8 (3)
C36—C18—C24—N2	119.8 (2)	C9—C8—N1—C5	171.0 (2)
C17—C18—C24—N2	−121.96 (17)	C28—C24—N2—C23	33.7 (3)
C19—C18—C24—N2	−1.5 (2)	C18—C24—N2—C23	−100.4 (2)
C36—C18—C24—C28	−17.0 (3)	C25—C24—N2—C23	150.5 (2)
C17—C18—C24—C28	101.2 (2)	C28—C24—N2—C20	156.03 (18)
C19—C18—C24—C28	−138.27 (19)	C18—C24—N2—C20	21.9 (2)
C36—C18—C24—C25	−128.07 (19)	C25—C24—N2—C20	−87.19 (19)
C17—C18—C24—C25	−9.86 (19)	C22—C23—N2—C24	164.2 (2)
C19—C18—C24—C25	110.64 (17)	C22—C23—N2—C20	41.8 (2)
N2—C24—C25—O7	−19.2 (2)	C19—C20—N2—C24	−33.9 (2)
C28—C24—C25—O7	108.2 (2)	C21—C20—N2—C24	−156.53 (18)
C18—C24—C25—O7	−132.45 (18)	C19—C20—N2—C23	97.2 (2)
N2—C24—C25—O6	99.09 (18)	C21—C20—N2—C23	−25.5 (2)
C28—C24—C25—O6	−133.58 (17)	C3—C2—O1—C1	−2.6 (4)
C18—C24—C25—O6	−14.2 (2)	C7—C2—O1—C1	178.0 (3)
N2—C24—C25—C26	−139.87 (18)	C12—C11—O3—C9	−38.0 (3)
C28—C24—C25—C26	−12.5 (2)	C16—C11—O3—C9	144.0 (2)
C18—C24—C25—C26	106.85 (18)	C10—C9—O3—C11	94.6 (3)
O7—C25—C26—C35	65.5 (3)	C8—C9—O3—C11	−168.2 (2)
O6—C25—C26—C35	−58.2 (3)	O4—C36—O5—C37	−5.3 (3)
C24—C25—C26—C35	−173.8 (2)	C18—C36—O5—C37	174.0 (2)
O7—C25—C26—C27	−112.5 (2)	C8—C17—O6—C25	−168.15 (17)
O6—C25—C26—C27	123.8 (2)	C18—C17—O6—C25	−42.8 (2)
C24—C25—C26—C27	8.2 (2)	O7—C25—O6—C17	155.54 (17)
C35—C26—C27—C28	−178.5 (2)	C26—C25—O6—C17	−79.2 (2)
C25—C26—C27—C28	−0.2 (3)	C24—C25—O6—C17	35.3 (2)

### Hydrogen-bond geometry (Å, º)

**Table:**

*D*—H···*A*	*D*—H	H···*A*	*D*···*A*	*D*—H···*A*
C33—H33···O4^i^	0.93	2.49	3.409 (3)	173
C37—H37*C*···O7^ii^	0.96	2.53	3.217 (3)	128

Symmetry codes: (i) −*x*, −*y*, −*z*+1; (ii) *x*−1, *y*, *z*.
